# From Pixels to Patterns: A Multidimensional Framework to Decode Cytoskeletal Organization

**DOI:** 10.34133/csbj.0113

**Published:** 2026-06-30

**Authors:** Diogo Fróis Vieira, Joana Figueiredo, João Sanches

**Affiliations:** ^1^ Institute for Systems and Robotics (ISR), LARSyS, Instituto Superior Técnico (IST), 1049-001 Lisboa, Portugal.; ^2^ i3S - Instituto de Investigação e Inovação em Saúde, University of Porto, 4200-135 Porto, Portugal.; ^3^ IPATIMUP - Institute of Molecular Pathology and Immunology of the University of Porto, 4200-135 Porto, Portugal.; ^4^ Department of Pathology, Medical Faculty of the University of Porto, 4200-319 Porto, Portugal.

## Abstract

The cytoskeleton is a dynamic filamentous network that supports essential cellular processes, from shape maintenance to cell division and migration. Advances in microscopy and computational image analysis now enable visualization and quantification of its organization with increasing precision, either as a whole structure or at an individual filament level. However, existing approaches to describe cytoskeletal architecture remain constrained by network complexity and heterogeneity in imaging and processing methods. In most studies, analysis focuses on a single organizational parameter, providing valuable but limited insights into cytoskeletal behavior and thus lacking an integrated perspective. Herein, we have compiled and analyzed the quantitative metrics reported for cytoskeletal characterization in 2-dimensional microscopy images, and organized them into a structured framework that synthesizes current methodologies. This pipeline addresses 8 complementary aspects, including morphology, orientation, quantity, compactness/density, bundling/thickness, connectivity, complexity, and interaction with cellular organelles, with each aspect representing a distinct dimension of filament structure. By classifying descriptors from diverse studies into a coherent conceptual framework, this review unveils a foundation for systematic and comparable analyses of cytoskeletal organization. Further, it can guide future investigations, supporting a more consistent interpretation of cytoskeletal organization across biological systems and experimental contexts.

## Graphical Abstract

**Figure:**
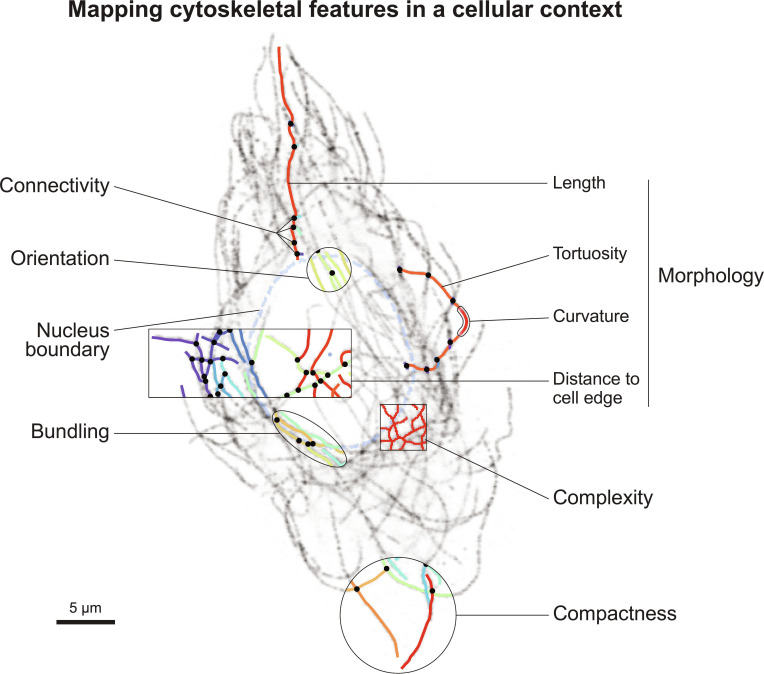

## Introduction

Filamentous formations are ubiquitous across biological and physical systems, spanning an extraordinary range of spatial scales. Examples include large-scale phenomena such as galaxies [1], solar filaments [2], and tree branches [3], as well as microscopic structures, including vascular networks [4], retinal layers [5], neuronal networks [6], and the cytoskeleton. These filaments exhibit organization patterns that determine their function and dynamics across scales.

In biological systems, filamentous structures form adaptable networks that combine mechanical and signaling functions. At the cellular level, for instance, the cytoskeleton is a prime example of such organized filamentous networks, presenting a dynamic, 3-dimensional (3D) scaffold composed of filament-forming proteins [7]. This structure is constantly remodeled to sustain cell shape, anchor organelles, and ensure mechanical resilience [8]. It also orchestrates essential processes such as intracellular transport, signaling, and cell-cell communication [8]. The cytoskeleton comprises 3 major filament systems—microtubules, actin filaments, and intermediate filaments—each with distinct architecture and mechanical properties [9]. Microtubules are the stiffest filaments, forming polarized tracks that span the cell and assemble into mitotic spindles during cell division [10], as well as into specialized structures like cilia and axons [11], acting as substrates for kinesin and dynein motors that generate directed forces for intracellular transport. Actin filaments, in turn, generate contractile forces in stress fibers through the action of myosin motors, or drive membrane protrusions in lamellipodia [12]. Intermediate filaments, which are made up of various proteins such as keratins, vimentin, or neurofilaments, connect different cellular regions and reinforce integrity without directional polarity [13].

Understanding the organization of the cytoskeleton increasingly relies on quantitative image analysis. Modern microscopy and computational image processing have enabled detailed descriptions of cytoskeletal architecture in diverse contexts, namely, drug-induced filament fragmentation [14], mechanically driven alignment [15], reorganization during epithelial–mesenchymal transition [16], and mitotic spindle formation [10]. However, existing studies usually focus on a single organizational aspect (filament length, alignment, or orientation), lacking an integrated framework that considers the multidimensional nature of cytoskeletal organization as a whole.

Typical image-based analyses follow 4 major steps: data acquisition, preprocessing, segmentation, and feature extraction [17,18]. While advances in microscopy yield increasingly heterogeneous datasets, differing in contrast, noise, and scale [18], these pipelines remain fragmented and are often tailored to specific imaging conditions. Importantly, despite many efforts to quantify cytoskeletal features, there is still no broad classification linking image-derived metrics to distinct organizational dimensions.

In this review, we aim to address this issue by systematically compiling quantitative descriptors of cytoskeletal architecture and categorizing them according to the organizational aspect they describe. Our goal is to provide a comprehensive basis for describing and comparing cytoskeletal organization across biological contexts by integrating existing methodologies into a robust framework.

## From Microscopy Images to Quantitative Descriptors of Cytoskeletal Organization

**Fig. 1.**
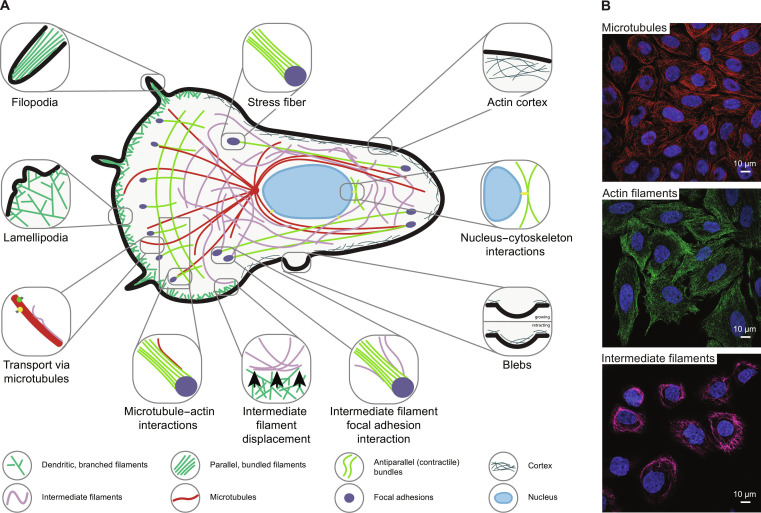
Cytoskeletal structures and filaments in human cells. (A) Schematic representation of filament organization and molecular interactions that shape cytoskeletal architecture. (B) Confocal microscopy images (pixel resolution, 0.08 μm) of cells from the Human Protein Atlas dataset. Microtubules (image 1169_H10_1; antibody HPA064019) and actin filaments (image 1339_D6_2; antibody HPA070614) are shown in the SiHa cell line, whereas intermediate filaments (image 1720_H1_12; antibody HPA069771) are marked in A-431 cells. Cells were fixed, permeabilized, and stained following the standard Cell Atlas protocol. Nuclei were counterstained with DAPI. Microtubules consist of α/β-tubulin heterodimers that self-associate into long, hollow, polarized tubes. Actin filaments are composed of globular actin (G-actin) monomers that assemble into thin, semiflexible, and polarized polymers. Intermediate filaments are built from diverse proteins such as keratins, vimentin, or neurofilaments and form flexible rope-like structures that confer mechanical stability.

Imaging is a fundamental tool in cytoskeleton research, providing the foundation for investigating its architecture and dynamics. It enables detailed visualization of cytoskeletal components and their spatial organization within the cellular environment (Fig. 1). Techniques such as fluorescence microscopy and cryo-electron tomography offer complementary advantages: fluorescence methods enable high-throughput imaging of dynamic processes but suffer from optical blur and limited resolution, whereas cryo-electron tomography achieves higher spatial resolution but requires fixed cells, yielding images with low signal-to-noise ratio and contrast [17,18]. Many conventional light microscopy techniques are fundamentally limited by diffraction, which imposes a resolution comparable to or larger than the diameter of individual filaments, precluding reliable filament-level visualization without the use of super-resolution modalities [17]. Beyond visual inspection, extracting meaningful information on cytoskeletal organization demands dedicated image preprocessing and segmentation workflows. Different cytoskeletal systems also present distinct imaging and analysis challenges, since actin filaments, microtubules, and intermediate filaments differ in their organization, curvature, thickness, and spatial arrangement, which influence the suitability of enhancement, segmentation, and descriptor-extraction strategies.

### Image preprocessing for signal optimization

**Fig. 2.**
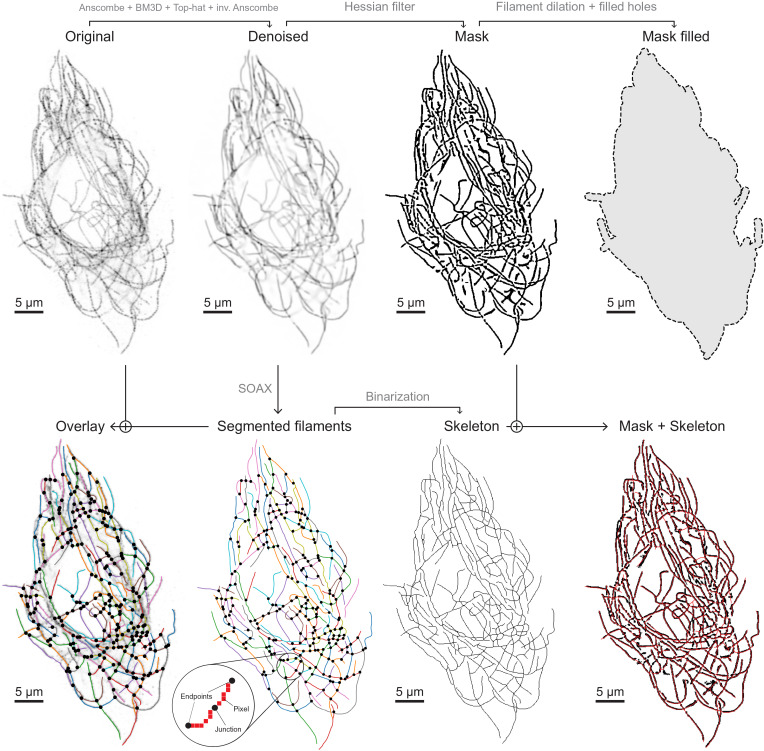
Preprocessing and segmentation of microtubules in a human cervical squamous carcinoma cell. Illustration of the image-processing workflow applied to a SiHa cell from the Human Protein Atlas dataset (pixel resolution, 0.08 μm). The illustrative workflow was implemented using SOAX along with custom image-processing and analysis scripts; the corresponding code is available through the following GitHub repository: https://github.com/diogojfv/HPACellCytoskeleton. The original microtubule image (1169_H10_1; antibody HPA064019) underwent a sequence of operations comprising classical Anscombe transform, BM3D denoising, inverse Anscombe, and white top-hat filtering for background removal. The resulting denoised image was processed with SOAX, which segments individual filaments into continuous pixel-coordinate paths, each displayed in a distinct color. Filament junctions and endpoints are indicated by black dots. For preservation of filament thickness information, a complementary cytoskeletal mask was created by binarizing the denoised image upon application of a Hessian-based filter. The total cytoplasmic area was then estimated by dilating and filling the holes of the binary mask. To generate the skeleton, the SOAX output was binarized. The mask was subsequently overlaid with the skeleton. For clarity purposes, image intensities were inverted.

Cytoskeletal structures can be modeled as networks of curvilinear objects, reflecting the filamentous geometry of the underlying proteins. The purpose of preprocessing is to enhance the visibility of these structures by separating filamentous signals from noise, blur, background, and other nonlinear artifacts [17]. Most current preprocessing methodologies rely on intensity-threshold-based approaches, usually encompassing denoising/deblurring and filament-enhancement stages. Common denoising techniques include Gaussian filters [15,17,19] and image deconvolution [20]. Filament-enhancement filters, such as Laplacian and directional Gaussian filters [15], linear and orientational filter transforms [20–22], Hessian matrix-based filters [23], and steerable filters [24,25], are frequently exploited after denoising to emphasize curvilinear or tubular geometries. Figure 2 illustrates a typical pipeline applied to microtubules of a human cervical squamous cell carcinoma cell (SiHa) obtained from the Human Protein Atlas dataset (Table 1). Denoising and background removal were essential to preserve filament intensity continuity, enabling accurate segmentation with SOAX, a software tool based on the Stretching Open Active Contours framework [26].

**Table 1.** Illustrative workflow of image processing. Summary of the main processing steps, associated algorithms, and key parameters used in the workflow applied to the microtubule image shown in Fig. 2.

Methodology step	Method	Parameter	Description	Value/Setting
Preprocessing	Gaussian filter	Sigma	Standard deviation for Gaussian kernel	0.1
	Classical Anscombe transform	N.A.	Variance-stabilizing transform for Poisson-like noise	N.A.
	BM3D	Sigma	Assumed noise level	0.2
		Profile	Noise setting	np profile
	Inverse Anscombe	N.A.	Inverse variance-stabilizing transform applied after denoising	N.A.
	White top-hat filtering	Sigma	Radius of disk structuring element	11
	Hybrid Hessian filter	Gamma	Sensitivity to areas of high variance/texture/structure	500
		Beta	Sensitivity to deviation from a blob-like structure	0.36
		Mode	Correction of values outside the image borders	Reflect
Filament tracing/instance segmentation	SOAX	Ridge threshold	Minimum ridge strength required to initialize and propagate snakes	0.005
		Gamma	Regularization/smoothness parameter controlling snake deformation	4
		Minimum snake length	Minimum accepted snake length	20
Cytoplasmic area estimation	Dilation + hole filling of binary mask	Dilation radius/size	Dilation with disk structuring element of the skeleton	6

N.A., not applicable

### Segmentation of filamentous structures

Following image enhancement, segmentation delineates cytoskeletal filaments from the background. Although segmentation is required for most descriptor classes, certain quantitative features, particularly those derived from grayscale intensity distributions or texture matrices, can be extracted directly from the raw or preprocessed image, bypassing explicit filament extraction entirely.

Early approaches typically aimed to generate binary representations of the cytoskeleton, either as global masks of the filamentous region or as thin one-pixel-wide centerlines, allowing intensity distribution analysis within the cytoskeletal area [27]. To this end, intensity-based thresholding has been widely employed. Thresholding can be applied globally, as in Otsu’s method [22,28]; adaptively, for instance, using median-based thresholds [14,17]; or through hysteresis strategies such as the Canny edge detector [29,30]. These procedures yield binary masks of filaments, which may undergo skeletonization to reduce the segmented objects to one-pixel-wide representations [13,19,31]. While skeletonization facilitates subsequent analysis, it often distorts filament geometry and fragments intersecting filaments, thereby introducing artifacts such as incorrect filament connectivity, artificial fragmentation at intersections, and misestimation of filament length [32]. Alternatives also include trainable pixel-classification approaches such as Ilastik, as well as semiautomatic tracing tools guided by user-defined seeds, such as JFilament, SNT, or BigTrace, which can be particularly useful in challenging datasets where fully automatic segmentation remains unreliable [33–35].

In an attempt to individualize filaments, a process commonly referred to as instance segmentation, several methods apply straight lines or line segments to approximate cytoskeletal proteins, given their quasi-straight geometries. Extraction of these segments can be performed semiautomatically using the Hough Transform, Line Segment Detector [36], or Linear and Orientation Filter Transforms [21]. These methods result in a set of lines distributed throughout the cytoplasm, representing individual fibers with defined positions, orientations, and lengths [15]. However, this approximation may fail when filaments display curvatures or deviate from linear geometry, or when postprocessing steps such as line merging are omitted. Although linear approximations have been employed in earlier works, they are only suitable for relatively stiff, straight filaments, and frequently fail to accurately describe filament geometry in realistic cellular contexts. As a result, they are being superseded by more flexible representations. Beyond linear approximations, individual filaments can be better represented as continuous pixel paths and the analysis may incorporate the intensity of each constituent pixel [37,38]. Among such methods, stretching open active curves (SOACs) constitutes a popular segmentation strategy for the extraction and quantification of filaments [13,26,33]. SOACs evolve dynamically from snake tips to delineate the central lines (ridges) of the filaments in the image, while the SOAX implementation outputs the cytoskeleton as a set of individual SOACs, each corresponding to a single filament [26] (Fig. 2).

More recently, segmentation pipelines have increasingly incorporated machine learning, either as a complement or a replacement to classical approaches [17]. Classical segmentation methods, while widely used, are often sensitive to noise, filament contrast variability, and complex cellular backgrounds, and typically require careful parameter tuning to perform reliably across different imaging conditions. Deep learning has been particularly transformative in bioimage enhancement and filament segmentation, with architectures based on U-Net, an encoder–decoder convolutional network originally designed for biomedical image segmentation, enabling highly accurate cytoskeletal imaging [39–41]. These methods overcome many limitations of conventional pipelines, offering improved robustness to noise, filament contrast variability, and complex cellular backgrounds. However, deep learning approaches have some limitations, as their performance is heavily dependent on the quality and diversity of training data.

Postprocessing strategies are often applied to refining segmentation outputs, merging fragmented filaments, resolving overlaps, and identifying filament tips or junctions [42]. Alioscha-Perez et al. [15] proposed a merging strategy that iteratively connects short, fixed-length line segments based on their overlap and alignment. The Python library Skan individualizes filaments from skeletonized objects, but overlapping structures remain a major limitation since intersections can truncate filaments into multiple shorter segments [37]. Zhang et al. [22] addressed this issue by merging network paths after skeletonization according to geometric constraints (similarity, proximity, and continuity), thus preventing artificial breaks. More recently, a modified ResNet, which is a deep convolutional network using residual skip connections to facilitate gradient flow and localization tasks, was explored to detect filament junctions and endpoints, mitigating the artifacts caused by skeletonization. Specifically, ResNet was trained for keypoint detection (endpoints and junctions) using an orientation/contrast-guided fast-marching algorithm, reconstructing continuous tracks while avoiding the spurious short branches introduced by skeletonization [32].

Distinguishing true filament cross-links from simple overlaps is also demanding, particularly when 3D structures are projected onto 2D. These effects can distort descriptors related to length, connectivity, branching, and topology. To mitigate these risks, visual validation and benchmarking against manual annotations should be combined with parameter choices tailored to the filament system, since actin, microtubules, and intermediate filaments differ in curvature, bundling, and cross-linking behavior. A comprehensive overview of software for cytoskeletal segmentation and analysis, including applicability to different filament systems and extractable parameters, is provided by Østerlund et al. [27] and summarized in Table 2.

**Table 2.** Summary of image-processing approaches used in cytoskeletal image analysis, grouped according to their role in the analysis pipeline. The table outlines common methods for denoising, filament enhancement, segmentation, and postprocessing, along with their main purpose and corresponding references.

Category	Approach	Purpose	References
Denoising	Gaussian filter	Reduces high-frequency noise and uniformizes the signal	[15,17,19]
	Image deconvolution	Corrects optical blur	[20]
Filament enhancement	Laplacian/Directional Gaussian filters	Enhances curvilinear structures	[15]
	Linear and orientational filter transforms (LFT/OFT)	Enhances oriented filament structures	[20–22]
	Hessian matrix-based filters	Detects tubular/curvilinear geometries	[23]
	Steerable filters	Extracts local orientation and enhances filaments	[24,25]
Binarization	Intensity thresholding (Otsu, adaptive, hysteresis/Canny)	Generates binary filament masks	[22,28–30]
	Skeletonization	Reduces masks to one-pixel-wide centerlines	[13,19,31]
	Stretching open active curves (SOACs)	Instance segmentation as continuous pixel paths	[26,33]
	U-Net (deep learning)	Robust filament segmentation	[39–41]
Segmentation and postprocessing	Line segment merging	Connects fragmented line segments	[15]
	Skeleton-based path merging (geometric constraints)	Prevents artificial filament breaks	[22]
	Skan (Python library)	Individualizes filaments from skeletonized objects	[37]
	ResNet keypoint detection + fast-marching	Detects junctions/endpoints; reconstructs continuous tracks	[32]

### Feature extraction and quantitative characterization

The choice of cytoskeletal representation fundamentally determines which quantitative features can be extracted and what aspects of organization can be described. Spatial grayscale distributions, line segment rearrangements, or adjacent pixel paths provide distinct computational frameworks for handling cytoskeletal data. Each is defined by a distinct mathematical basis from which quantitative features can be extracted to better understand cytoskeletal organization. A feature is hence a measurable property of a structure that conveys information ranging from the global distribution of cytoskeletal components within a cell to the specific length, orientation, or curvature of an individual filament. Different analytical methods produce different types of features, yet they often converge in describing similar organizational aspects. For instance, a long filament may be approximated to a straight line with defined endpoints and orientation, or, more precisely, to a continuous pixel path whose coordinates and intensities capture local geometry and signal variation.

Grayscale-based analyses have been widely reported in the literature [19,43,44]. Morphological features, such as radius, area, perimeter, centroid, or circularity, and intensity features, including coefficient of variation, standard deviation, mean intensity, and texture measures derived from gray-level co-occurrence matrices (GLCMs), are commonly extracted in this type of analysis [10,43,45]. However, the complex topography of the cytoskeletal meshwork limits the interpretation of these descriptors, as they do not distinguish individual filaments and therefore cannot accurately describe cytoskeletal organization. Alternatively, cytoskeletal structures can be represented as collections of line segments, enabling the study of filament length and orientation distributions [15,30]. This abstraction facilitates global characterization of filament alignment and anisotropy, but lacks information regarding local curvature or filament thickness. A more detailed representation is achieved when filaments are traced as continuous pixel paths, preserving both geometry and intensity data. Beyond the level of individual filaments, the cytoskeleton can also be modeled as a network, where filaments constitute edges and their intersections form nodes. This network representation enables quantitative assessment of connectivity, branching and alignment, providing complementary insights into cytoskeletal organization. Several studies have explored these distinct representations to extract features and investigate a variety of biological processes, namely, stress fibers or microtubule alignment, cytoskeletal–nucleus coordination, and organelle transport regulation [14,16,46,47].

## Quantitative Dimensions of Cytoskeletal Organization

Actin filaments, microtubules, and intermediate filaments form intricate and interdependent networks that continuously reorganize to support essential cellular functions, including migration and invasion, intracellular transport, mitosis, and mechanotransduction [8]. To fulfill these roles, the cytoskeleton dynamically adapts its architecture, with filaments varying in length, orientation, density, and connectivity according to the cellular context. In this sense, cytoskeletal organization refers to the spatial distribution of filaments and their interactions within the cytoplasm, encompassing both global and individual characteristics that evolve during cellular processes.

Because of its inherent complexity, cytoskeletal organization must be considered from multiple quantitative perspectives. In the field of image-based analysis, several studies have defined such perspectives and proposed corresponding quantitative descriptors. Kimori et al*.* studied and quantified the thickness, orientation, and complexity of cytoskeletal structures in plant cells, whereas others have described 4 organization aspects: orientation, parallelism, bundling, and density [19,48]. This approach was later extended to 3D, introducing node, segment, and connection descriptors to characterize filament networks as a whole [38]. Other reports describe cytoskeletal organization systematically in 5 categories, including bundling, connectivity, branching, directionality, and density [49]. More recently, Akenuwa et al*.* [50] proposed density, orientation, ordering, and bundling as universal parameters for reporting actin network organization. Although valuable, existing studies remain fragmented without a shared conceptual foundation, with each work defining its own set of organizational aspects. This heterogeneity hampers comparison across studies and limits the establishment of general principles. To address this gap, we propose an integrative framework that categorizes cytoskeletal organization into 8 complementary aspects, encompassing morphology, orientation, quantity, compactness/density, bundling/thickness, connectivity, complexity, and interaction with other organelles (Table 3). This framework enables consistent mapping of quantitative features to well-defined organizational dimensions, providing a comprehensive description of cytoskeletal architecture across biological contexts. Spatial distribution remains an important interpretative layer in the analysis of cytoskeletal organization, although it was not defined as a separate category since it can be captured through combinations of morphology, density, orientation, and organelle-relative descriptors.

**Table 3.** Quantitative descriptors of cytoskeletal organization can be grouped into 8 complementary organizational aspects. The table summarizes the principal metrics reported in the literature for each organizational aspect, the type of organization they capture, whether prior segmentation is typically required, their applicability to 2D or 3D data, and relevant references. All descriptors listed are presented in the context of 2D static microscopy image analysis.

Organizational aspect	Biological meaning	Metrics	Segmentation required	References
**Morphology**	Geometry and shape of individual filaments or of the whole cytoskeletal network	Filament length; geodesic length; curvature; tortuosity; Menger curvature; pixel-wise discrete curvature; mean signed curvature; mean absolute curvature; distance to cell edge/periphery; proportions of short/medium/long filaments; coefficient of variation of filament lengths; area; perimeter; volume; shape-similarity via curve-clustering algorithms	Partial. Length and curvature require segmentation; area and shape descriptors need only a binary mask	[14,15,22,28,29,32,37,38,45,52–56]
**Orientation**	Directional arrangement and alignment of filaments relative to a reference axis or point	Filament angle distribution; slope/segment orientation; orientation relative to horizontal axis; orientation relative to cell major axis; entropy-based multi-orientation index; local orientation from neighborhood angular distribution; alignment/anisotropy measures; partial actin-cytoskeletal deviation index (PAD); total actin-cytoskeletal deviation index (TAD); orientational order parameter (OOP); circular variance; local orientation anisotropy tensor/eigenvalue-difference metric; radiality/radial orientation score	Partial. Slope/segment-based and graph-based methods require segmentation, but pixel-based directionality (neighborhood angular distribution) and steerable filter approaches work on grayscale directly.	[13–16,19,22,24,30,47–49,57–59]
**Quantity**	Total amount of filamentous material, without spatial normalization	Average actin intensity (AAI); average microtubule intensity (AMI); mean grayscale intensity; total number of filaments; filament count from line segments; filament count from pixel paths	Partial. AAI/AMI and mean grayscale intensity = No segmentation. Filament count = Yes.	[15,30,31,54,55,60,61]
**Compactness/Density**	How tightly filament material fills space, and how much material exists per unit area/volume	Compactness as node distribution around center of gravity relative to surface; pore area; pore edge length; pore circularity; area fraction/filament area divided by total cell area; skeleton area fraction; density = filament pixels/total pore area; occupancy; linear density	Partial. Pore area/circularity require segmented masks. Pixel occupancy and area fraction require only a binary mask, not individual filament tracing.	[28,48,49,52,63,64]
**Bundling/Thickness**	Degree of filament bundling and apparent filament thickness	Intensity histogram skewness; coefficient of variation of intensity; pattern spectrum median thickness; width maps; local width from distance between intensity minima; width = object area/skeleton length; EDT-based width/thickness; width from highest mean EDT gradient; mean EDT along skeleton; average filament diameter = 4× mean EDT distance; average filament diameter = 2× EDT on skeleton pixels	Partial. Intensity histogram skewness and CV = No segmentation needed. Pattern spectrum, EDT-based width, skeleton-based width = Yes.	[19,22,28,29,48,49,61,64,67,68]
**Connectivity**	Local inter-filament linking and global network topology/robustness	Connectivity class/type 0, type 1, type 2, type 3 filaments; number of junctions; ratios of connectivity types (e.g. type 2/type 3 *vs*. type 1); mean filament-junction angle; number of crossings; algebraic connectivity (second-smallest Laplacian eigenvalue); assortativity	Yes. All connectivity metrics require individual filament segmentation and skeletonization.	[14,37,38,49,64]
**Complexity**	Structural irregularity, disorder, and multiscale intricacy	Fractal dimension; box-counting fractal dimension; local fractal dimension of image regions; global fractal dimension; Shannon entropy; GLCM-based entropy	Partial. Fractal dimension can be computed from grayscale images, binary masks, or skeletons, depending on implementation; entropy-based metrics require no segmentation.	[69–73]
**Interaction with other organelles**	Spatial and functional coupling between the cytoskeleton and organelles, especially the nucleus	Nuclear volume; nucleus–cytoskeleton centroid distance; spatial position of cytoskeletal structures relative to the nucleus; nucleus/cytoskeleton area ratio; texture analysis of cortical actin ruffling relative to nucleus/cell regions; vimentin architecture classes relative to nuclear position	Usually yes, including segmentation of both the cytoskeleton and the organelle	[43,47,76–78]

### Morphology

The morphology of the cytoskeleton refers to the geometry and overall shape of its structures at the filament scale or at the whole cytoskeletal network. The cytoskeleton comprises 3 main classes of fibers, microtubules, actin filaments, and intermediate filaments, which differ in mechanical stiffness, polarity, dynamic (dis)assembly and association with specific molecular motors (Fig. 1) [51]. These biophysical properties give rise to distinct morphologies as seen in microscopy images. Microtubules generally appear as quasi-straight filaments, actin filaments as string-like paths, and intermediate filaments as curved or contorted fibers.

**Fig. 3.**
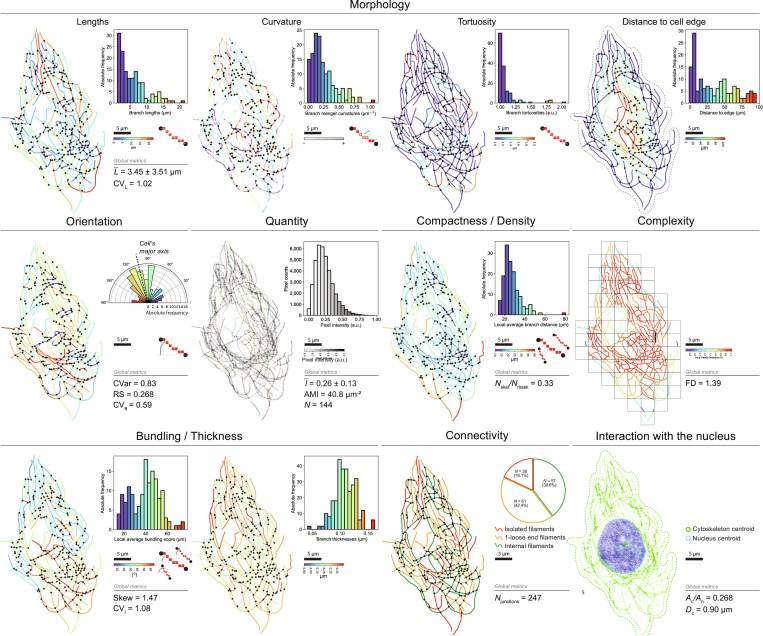
Quantitative description of microtubule organization in a human cervical squamous carcinoma cell. Cytoskeletal organization metrics were extracted from an image of a SiHa cell stained for microtubules from the Human Protein Atlas dataset (image 1169_H10_1; antibody HPA064019). The analysis is presented as an illustrative example of how representative descriptors map onto the proposed organizational aspects. It was performed using SOAX along with custom Python scripts for feature extraction, quantification, and visualization; the corresponding code is available through the following GitHub repository: https://github.com/diogojfv/HPACellCytoskeleton. Histograms show the distributions of filament-level features, while global metrics were computed at the whole-cell level (mean ± standard deviation). In the top panel, morphology was evaluated by computing filament length, curvature, tortuosity, and distance to the cell edge. The global metrics shown are the mean filament length $\bar{L}$ and coefficient of variation of the length distribution ${\mathrm{CV}}_{L}$. The cytoskeleton images (left) were multiplied by the segmentation mask to obtain intensity distributions and statistical features. The graphs (right) depict each of the 144 microtubule filaments segmented by SOAX assigned to a unique color. Orientation, displayed in the middle panel, was quantified relative to both the horizontal axis and the cell’s long axis, and the corresponding distributions were extracted; the corresponding global metrics are the circular variance CVar and radial score RS of angle dispersions based on the horizontal axis, and coefficient of variation based on the cell’s long axis ${\mathrm{CV}}_{\theta }$. Quantity was evaluated by the total number of filaments N as well as by the distribution of signal intensity within cytoskeletal pixels, shown in the histogram, or by quantitative metrics derived from these data, including mean ± standard deviation intensity and the angular mean intensity (AMI). Local density is represented by coloring filaments according to their mean distance to neighboring filaments. Complexity is illustrated by box-counting analysis in 48 × 48 patches of the filament network, and summarized by the fractal dimension FD. Bundling (bottom panel) is represented as the mean angular difference between neighbors and estimated using the global metrics skewness Skew and coefficient of variation ${\mathrm{CV}}_{I}$ from the intensity distribution. Thickness corresponds to twice the mean Euclidean Distance Transform (EDT) along each filament, and connectivity level is depicted by coloring filaments according to the number of links with adjacent filaments and counting the number of junctions $N_{\text{junctions}}$. In the final panel, the nucleus channel is overlaid with the microtubule network, and features such as nucleus/cytoskeleton area $A_{C}/A_{N}$ and distance between nuclear and cytoskeletal centroids $D_{C}$ can be computed from the respective segmentation masks. Pixel resolution: 0.08 μm.

At the filament level, length is the most widely used morphological descriptor. Changes in filament length are often associated with cytoskeletal remodeling, for example, during the extension of protrusive structures in migrating cells or following drug-induced depolymerization, fragmentation, or other disruptions of cytoskeletal integrity [29]. Length can be obtained either from line-segment representations or from adjacent pixel-path reconstructions of filament networks [15,22,52]. In the former case, it corresponds to the Euclidean distance between endpoints, whereas in the latter case, length is estimated by summing the distances between consecutive pixel coordinates along the filament (Fig. 3). Alternative approaches, such as the use of geodesic maps derived from segmented masks, have been proposed to avoid artifacts introduced by skeletonization [32].

Curvature and tortuosity describe how filaments twist or bend in space. These descriptors are also related to filament persistence length, a classical measure of bending rigidity widely used in cytoskeletal biology. Filaments with larger persistence lengths tend to remain straighter over longer distances, whereas those with lower persistence lengths exhibit greater bending [51]. Curvature measures the local or global bending of a filament at a given point or along its length, whereas tortuosity captures the overall windiness of a filament path relative to its end-to-end distance. Highly curved or winding fibers exhibit greater tortuosity, a metric commonly adopted from cardiovascular imaging, where it is used to detect arterial abnormalities [53]. Graph-based cytoskeletal representations have explored this descriptor to quantify filament contortion [14,37]. Other curvature metrics include the Menger curvature, which defines the radius of the circumcircle formed by the start, the end, and the most distant point along a filament, as well as pixel-wise discrete curvature calculations that reveal local bending heterogeneity [38,54]. FilamentSensor 2.0, for example, represents filaments as polylines and computes mean signed and mean absolute curvature from the cumulative turning angles, providing a global measure of bending per unit length [55]. Some studies also analyzed how filament distribution varies with distance from the cell periphery [54].

At the cell or network scale, morphological analysis seeks to capture comprehensive structural properties. Filaments can be categorized into short, medium, and long classes [28], and the coefficient of variation of filament lengths has been used to assess dispersion within the network [14]. Classical morphology-based descriptors, including area (2D), volume (3D), or perimeter, are typically extracted from the segmented cytoskeletal masks [38,45,54]. Recently, innovative approaches such as curve-clustering algorithms have been introduced to group filaments based on shape similarity, allowing higher-level morphological classification [56].

### Orientation

Orientation represents the angular alignment of filaments relative to a reference axis, such as the horizontal axis, the cell’s long axis, or nearby structures and organelles [48]. It describes not the position of the filaments in space, but rather the direction in which they are oriented. Across the cell, the collection of filament orientations forms an angle distribution, whose spread increases with the degree of angular disorder [16]. Organized orientation patterns can exhibit parallel, antiparallel, or even radial arrangements. Parallelism expresses how narrowly filament orientations cluster around a local mean direction, with higher parallelism corresponding to greater alignment [48]. Of note, in static microscopy, it is generally not possible to identify the (+) or (−) ends of filaments, making it difficult to distinguish between parallel and antiparallel conformations. In contrast, radial configurations are defined by the convergence or divergence of filaments around a point (e.g., the centrosome), from which microtubules emanate centrifugally [57,58].

Efforts to quantify filament orientation have been extensively reported in the literature. For instance, entropy-based metrics have been used to characterize global orientation distributions, known as the multi-orientation index, derived from morphological openings applied to segmented cytoskeleton masks [19]. In qualitative terms, entropy expresses the degree of disorder of the orientation distribution: low entropy indicates that filaments are concentrated around preferred directions, whereas high entropy indicates a broader, more disordered distribution. In this sense, this measure distinguishes cells with anisotropic or random filament orientation profiles from those with homogeneous alignment [19]. Other methods estimate orientation directly from filament representations. Line-segment models quantify orientation through the slope of each segment [15], while graph-based frameworks evaluate the dispersion of edge angles relative to the cell’s major axis [14]. Pixel-based techniques can also infer local orientation through analysis of the angular distribution within a defined window around each pixel and subsequent estimation of neighborhood directionality [22]. To assess higher-order organization, several metrics capture local or global alignment and anisotropy. In steerable-filter approaches, local filament orientation is obtained as the angle that maximizes the response of an oriented filter bank at each pixel, yielding dense orientation fields that can be translated into alignment or order parameters [13,24]. Order parameters are descriptors of how strongly filaments share a common orientation, with higher values generally corresponding to greater orientational coherence. Other examples comprise the partial and total actin-cytoskeletal deviation indices, designed to quantify actin parallelism in fibroblasts under inhibitory treatment [30], and the orientational order parameter (OOP), which describes the alignment of actin stress fibers and helps identify intermediate epithelial-to-mesenchymal transition states [16]. This concept is closely related to nematic order, which is commonly used to describe filament organization when orientations are considered equivalent under 180° reversal. Conceptually, the OOP reflects the extent to which filaments adopt a common axis of orientation, ranging from disordered arrangements to highly aligned ones.

Circular statistics have also been employed, with the circular variance of orientation angles used to evaluate the spread of collagen or filament alignment [59]. Accordingly, circular variance can be understood as a measure of angular spread, with low values indicating tightly clustered orientations and high values indicating broad angular dispersion. Furthermore, an index of local orientation anisotropy was proposed, in which each filament segment contributes a direction vector forming a rank 2 tensor, averaged over a circular neighborhood and weighted by filament length. The dominant eigenvector gives the main orientation, while the eigenvalue difference quantifies anisotropy [49]. Although radial organization has been less explored in cytoskeletal studies, recent work from our group provided a quantitative approach to this aspect [47]. By analyzing the angular distribution of microtubules segmented as line segments, we identified the cytoplasmic point where radiality reaches its maximum, offering a robust framework for quantifying this otherwise overlooked organizational pattern.

### Quantity

Quantity assesses how much filamentous material occupies a given cellular region. It is a straightforward measure that reflects filament abundance without considering their spatial arrangement, orientation, or distribution. Variations in filament quantity depict the balance between polymerization and depolymerization, and may therefore accompany changes in cellular state, including proliferation, quiescence, or adaptation to mechanical and biochemical cues [12].

The estimation of quantity as an aspect of organization has been addressed in a versatile manner, depending on data availability. When segmentation of individual filaments is not feasible, grayscale intensity distributions are often used as indirect measures of filament quantity. For example, average intensity metrics such as the average actin intensity and average microtubule intensity have been derived from grayscale histograms to estimate filament content [30,60].

When individual filaments can be segmented, quantity can be directly assessed by counting the total number of detected filaments, a strategy widely adopted in the field [55,61]. This can be achieved using representations based on line segments or adjacent pixel paths, each providing a discrete yet complementary measure of filament abundance within the cell [15,31,54].

### Compactness and density

While quantity reflects the total amount of filamentous material, it does not account for how that material is spatially arranged within a given volume. When filament positioning is considered, an equal number of filaments may occupy the available space in markedly different ways, giving rise to regions that vary in density and compactness. In uniformly distributed networks, filaments may efficiently fill the available space. In contrast, when filaments cluster within specific areas, they form compact or densely packed subregions. Although compactness and density are related, they describe distinct organizational perspectives [48,62]. Compactness reflects how much empty space exists within a structure, with a more compact arrangement leaving less void inside [63]. Density, on the other hand, quantifies the amount of filamentous material per unit area or volume.

Compactness in 3D cytoskeletal networks has been estimated by measuring how closely nodes are distributed around the network’s center of gravity relative to its surface, expressed as a normalized distance difference [64]. The same concept has been approached indirectly by analyzing pore geometry, commonly referred to as mesh size, treating the voids between filaments as negative spaces [63]. Indeed, features such as pore area (or mesh size), edge length, and circularity have been extracted from segmented pore networks to characterize compactness [52,63].

Density has often been computed as a ratio between the filamentous area and the total cellular area, using either the skeletonized or original segmentation mask [28,48]. Some analyses incorporate pore information, defining filament density as the total number of pixels assigned to the filamentous network divided by the total pore area [52]. A detailed strategy was established by Li et al*.* [49], which estimated density through 2 complementary metrics: occupancy, defined as the fraction of positive pixels within the binary image mask, and linear density that quantifies how much space is occupied by the length of skeletonized filaments. The latter is calculated by convolving the skeleton image with a directional 3 × 3 kernel and normalizing to the total number of pixels, thus capturing directional variations in filament packing [49]. Although these approaches differ in their mathematical formulation, they share the common goal of normalizing filament content to a spatial reference, distinguishing them from simpler measures such as filament number or total filament length. Pixel occupancy and area-based ratios provide global estimates of how much of the cellular space is occupied by filamentous material, pore-based measures capture density indirectly through the geometry of empty spaces, and skeleton-based metrics offer directionally sensitive estimates of filament packing that can reveal local heterogeneity within the network.

### Bundling and thickness

While compactness and density describe how filamentous material is spatially distributed across the cellular area, bundling and thickness estimate the lateral aggregation of individual filaments into multi-filament assemblies, reflecting a distinct organizational property that is not captured by spatial filling alone. The formation of tightly aligned filament assemblies plays a crucial role in the mechanical stability and functionality of the cytoskeleton. When multiple filaments agglomerate into compact arrays, they can collectively withstand mechanical stress and reinforce cellular integrity [12]. Such structures are key to diverse processes, namely, the bundling of actin filaments into protrusive formations (lamellipodia and filopodia) that drive motility, and the organization of microtubule bundles that form mitotic spindles during cell division or axonal arrays in cilia and flagella [10,65].

A persistent challenge in the quantitative study of bundling lies in resolving individual filaments within bundles. The lateral dimensions of microtubules (≈24 nm) and actin filaments (≈7 nm) are far below the resolution limits of common optical techniques like fluorescence microscopy [17,66]. Consequently, bundles often appear in images as single, thicker filaments, obscuring individual boundaries. Because of this limitation, filament thickness is typically analyzed as a proxy for bundling level. It should be noted, however, that apparent increased filament thickness may not exclusively reflect true bundling, as it can also arise from overlapping filaments that are not physically associated, or from imaging artifacts such as optical blur and out-of-focus signal. This approximation should therefore be interpreted with caution, particularly in dense networks or under suboptimal imaging conditions.

Quantitative approaches to evaluate bundling and thickness can be broadly grouped into 4 methodological categories. First, intensity-based methods estimate bundling indirectly from fluorescence intensity distributions. When filaments aggregate into bundles, the corresponding regions exhibit higher fluorescence, shifting the intensity histogram toward brighter values and increasing its asymmetry [67]. Statistical measures such as skewness, which quantifies the asymmetry of a distribution [48,49], and the coefficient of variation, which measures the relative dispersion of intensity values around the mean [68], have therefore been used to quantify bundle formation based on signal heterogeneity. Second, morphological approaches operating on binary masks apply pattern spectrum analysis to estimate global filament width. This pattern spectrum is obtained by sequential opening and closing operations with structuring elements of different sizes, whose median value reflects the average thickness [19]. Width maps derived from preprocessed masks have been proposed as an additional representation of filament diameter [61]. Third, grayscale-based estimation measures local filament width directly on grayscale images by tracing centerlines and computing, at each point, the distance between intensity minima on either side of the fiber, as implemented by Rogge et al. [29]. Fourth, skeleton and distance-transform approaches determine filament width as the ratio between the object area (number of pixels) and the number of skeleton pixels [28]. Other strategies rely on the Euclidean distance transform image (EDTI) to measure distances between filaments and their boundaries [22,49,64]. Zhang et al*.* obtained gradients along distance levels, and the distance with the highest mean gradient indicates the filament’s width, defined as twice this value [22]. Mean EDTI values along skeleton paths can also provide average filament thickness [64]. More recently, average filament diameter has been computed either as 4 times the mean EDTI distance, or as twice that value, when restricted to skeleton pixels [49]. Future developments in this area may combine local measures of parallelism and compactness, enabling the simultaneous quantification of filament alignment and bundling within subcellular regions.

### Connectivity

The structural cohesion of the cytoskeleton depends on interactions between individual filaments and within the network. These physical connections are mediated by cross-linking proteins, adhesion molecules, or direct filament branching, which define the degree of connectivity and influence network stability and mechanical properties. At the local scale, connectivity can be described by the number of junctions each filament establishes with its neighbors. A fully isolated filament forms no terminal contacts (type 0 connectivity), whereas partially connected filaments link to one or more partners at a single end (type 1). Filaments connected at both ends (type 2 or type 3) occupy more integrated positions within the network [37,38]. Aggregating these local patterns across all filaments provides an overview of the network’s branching architecture and its level of interconnection.

Quantitative analysis of connectivity requires individual filament segmentation, usually followed by connected component analysis on the corresponding skeletonized network [14,37]. Ratios between filaments of different connectivity types, type 2/type 3 versus type 1, are used to describe the degree of filament interlinking within the network [49,64]. Three-dimensional reconstructions further enable measurement of junction geometry, including the mean angles between filaments, specifically at connecting points [64]. These filament-based metrics describe local connections of individual filaments, capturing the immediate neighborhood of each filament within the network.

Graph-based approaches provide complementary metrics to assess global network topology and robustness. In actin networks, for example, the number of filament crossings and the second-smallest eigenvalue of the Laplacian matrix have been used to quantify algebraic connectivity, an indicator of overall structural robustness [14]. To infer connectivity heterogeneity, assortativity measures whether adjacent nodes tend to connect to others with similar degrees [14].

### Complexity

The complexity of the cytoskeleton captures the degree of heterogeneity and structural intricacy present across multiple spatial scales. This property can arise from variations in filament morphology, orientation, or connectivity, yet its usage as an independent organizational aspect remains debatable. When defined relative to these descriptors, complexity becomes inherently dependent on them. Nevertheless, specific mathematical formalisms, particularly those grounded in fractal geometry, have been widely adopted to describe the overall intricacy of cytoskeletal networks, supporting its treatment as a distinct category [69–71].

Fractals are self-similar geometric patterns characterized by noninteger dimensions that encode detail across scales [72]. The fractal dimension provides a statistical index of complexity by quantifying how structural detail changes with scale. Among available approaches, the box-counting method is a widely used algorithm to calculate the fractal dimension of cytoskeletal structures from grayscale images [70,72]. In this technique, an image is overlaid with grids of progressively smaller squares, and the number of boxes containing filament pixels is recorded. The slope of the resulting log–log relationship yields the fractal dimension, which increases with structural irregularity. A higher fractal dimension indicates a more intricate, space-filling network, whereas a lower fractal dimension is indicative of sparser, more ordered arrangements linked to cytoskeletal reorganization during differentiation or disease progression [72]. Studies have measured either the fractal dimension of discrete image regions to assess spatial variation [70], or the computed global values by averaging actin networks rotated at fixed angular intervals relative to the nucleus’ major axis [72].

A complementary perspective on cytoskeletal complexity is provided by entropy metrics, which quantify the level of disorder or unpredictability in filament organization. In this setting, entropy reflects how diverse or uncertain the structural pattern is, rather than the amount of filament material itself. Derived from information theory, Shannon entropy measures the average information content of an image, with higher values indicating greater structural variability and less predictable data, while lower values anticipate more uniform, ordered arrangements. Thus, low entropy corresponds to more repetitive or homogeneous patterns, whereas high entropy indicates richer local variation and less predictable organization. Entropy can be extracted from the image’s grayscale histogram or computed from GLCMs, which capture local intensity relationships and textural diversity across the cytoskeletal network [73].

### Interaction with other organelles

The cytoskeleton maintains physical and functional connections with various organelles, coordinating their positioning, transport, and mechanical behavior. Among these, the nucleus is the most studied, due to its large size, central localization, and key role in mechanobiology [74]. Indeed, it is the stiffest intracellular component and frequently limits cellular deformation during confined migration and invasion [75]. The dynamic coupling between the cytoskeleton and the nucleus occurs through force transmission across the nuclear envelope, linking filament organization to nuclear function [75].

Quantitative analyses have characterized this interaction using descriptors such as nuclear volume, centroid distance, and the spatial location of cytoskeletal structures relative to the nucleus, demonstrating that cytoskeletal and nuclear architectures undergo concomitant reorganization during *in vitro* invasion of cancer cells [47,76,77]. In this scope, a combined approach of marker-controlled watershed segmentation of nuclei and cytoplasm with texture analysis of the cortical actin “ruffling” region revealed specific cytoskeletal changes associated with tumor cell migration and cell–extracellular matrix adhesion [43]. Another study by Feliksiak et al*.* [78] classified “classic” versus “nutshell” vimentin architectures on different polymer substrates through quantification of vimentin signals, establishing a correlation between perinuclear intermediate filament organization and nuclear position. Although this coupling has been investigated in detail, cytoskeletal interactions with other organelles, namely, mitochondria, endoplasmic reticulum, or vesicles, remain much less explored and offer the ground for future studies.

## Future Perspectives

Quantitative image analysis has transformed the study of cytoskeletal architecture, enabling the extraction of diverse metrics that describe its organization from local filament geometry to whole-cell structure. Advances in image-processing workflows, including denoising, segmentation, and feature extraction, have progressively improved accuracy and reproducibility, while recent developments in deep learning promise further gains in robustness and automation [32]. These methodological refinements support the establishment of systematic frameworks capable of integrating heterogeneous metrics into coherent models of cytoskeletal organization.

The 8 organizational aspects discussed herein (morphology, orientation, quantity, compactness/density, bundling/thickness, connectivity, complexity, and organelle interaction) represent complementary dimensions through which cytoskeletal organization can be quantified. Collectively, they capture the multiple ways in which filament networks adapt during biological processes such as cell migration, division, polarization, or mechanotransduction [12]. Several examples demonstrate how orientation guides epithelial polarity, bundling assists protrusive activity, connectivity orchestrates trafficking, and complexity reflects structural disorder. Yet, important gaps remain surrounding interactions between the different filament systems and their coupling to organelles beyond the nucleus.

As automated analytical tools become increasingly accessible, awareness of common pitfalls is essential. Image quality directly determines the feasibility and accuracy of filament segmentation: as individual filaments become more resolvable, their extraction becomes more reliable and less dependent on error-prone steps such as skeletonization, which should be avoided in 2D analysis. Super-resolution modalities are particularly valuable in this regard, though access remains limited in most laboratories, where conventional setups require careful processing choices to mitigate their inherent limitations. Three-dimensional imaging of the cytoskeleton presents additional challenges, including poor axial resolution, slow acquisition speeds, and cell drift, hampering a reliable volumetric reconstruction of live cells. Maximum intensity projections are widely used as a workaround, but collapse spatial information, obscure true filament crossings, and introduce ambiguity in junction detection. Since the cytoskeleton is inherently 3D, future efforts should prioritize imaging approaches that faithfully capture its full architecture, such as light-sheet microscopy and adaptive optics, combining high lateral resolution with sufficient temporal resolution to follow dynamic reorganization in living cells. Broader adoption of quantitative cytoskeletal analysis will also depend on the accessibility and adaptability of tools. User-friendly platforms such as Ilastik, JFilament, SNT, BigTrace, SOAX, Skan, FilamentSensor, or ILEE provide different levels of support for segmentation, tracing and descriptor extraction, but their applicability varies with filament type, image quality, dimensionality, as well as with the specific organizational aspect under study. Open-source implementations, clearer reporting of parameter choices, and reusable analytical pipelines will be essential to improve reproducibility across laboratories and imaging conditions.

It should also be noted that the example presented here is limited to a single cell type and filament system. Cytoskeletal organization differs substantially across cellular models, and segmentation and feature extraction approaches need to be adapted accordingly, since methods optimized for one cell type or imaging condition do not necessarily apply to others. Systematic validation of these descriptors across diverse cellular systems, including epithelial cells, neurons, and cancer cells representing distinct cytoskeletal architectures, remains an important direction for future work. Alongside these efforts, standardizing feature definitions and extending analyses to 3D data will be critical to achieving a comprehensive and reproducible representation of cytoskeletal architecture. The present review outlines a conceptual basis for reliable quantitative descriptors, offering a path toward a consistent understanding of filamentous organization in health, disease, and development.
